# A Case-Control Study on Intimate Partner Violence during Pregnancy and Low Birth Weight, Southeast Ethiopia

**DOI:** 10.1155/2015/394875

**Published:** 2015-12-20

**Authors:** Habtamu Demelash, Dabere Nigatu, Ketema Gashaw

**Affiliations:** ^1^Department of Public Health, College of Medicine and Health Sciences, Madawalabu University, Goba, Bale, Ethiopia; ^2^Department of Nursing, College of Medicine and Health Sciences, Madawalabu University, Goba, Bale, Ethiopia

## Abstract

*Introduction*. Violence against women has serious consequences for their reproductive and sexual health including birth outcomes. In Ethiopia, though the average parity of pregnant women is much higher than in other African countries, the link between intimate partner violence with low birth weight is unknown.* Objective*. The aim of this study was to examine the association between intimate partner violence and low birth weight among pregnant women.* Method*. Hospital based case-control study was conducted among 387 mothers (129 cases and 258 controls). Anthropometric measurements were taken both from mothers and their live births. The association between intimate partner violence and birth weight was computed through bivariable and multivariable logistic regression analyses and statistical significance was declared at *P* < 0.05.* Result*. Out of 387 interviewed mothers, 100 (25.8%) had experienced intimate partner violence during their index pregnancy period. Relatively more mothers of low birth weight infants were abused (48%) compared with controls (16.4%). Those mothers who suffered acts of any type of intimate partner violence during pregnancy were three times more likely to have a newborn with low birth weight (95% CI; (1.57 to 7.18)). The association between overall intimate partner violence and LBW was adjusted for potential confounder variables.* Conclusion*. This research result gives insight for health professional about the importance of screening for intimate partner violence during pregnancy. Health care providers should consider violence in their practice and try to identify women at risk.

## 1. Introduction

The World Health Organization (WHO) defines violence against women as “the range of sexually, psychologically, and physically coercive acts used against women by current or former male intimate partners” [1]. It is related to violence of any kind that is likely to result in harm or suffering of women whether it occurs in private or in public [2].

Domestic violence is one of the most common forms of violence against women. It is the violence perpetrated by persons who have or had a relationship of kinship or affection with the woman and generally refers to the current or former male intimate partner [1, 2]. Intimate partner violence against women is a major worldwide epidemic which has been found in practically all societies [3]. According to WHO 2013 report, 1 in 3 women throughout the world will experience physical and/or sexual violence by a partner or sexual violence by a nonpartner [1]. Pregnancy may be a time of unique vulnerability to intimate partner violence (IPV) victimization because of changes in women's physical, social, emotional, and economic needs during pregnancy [1, 2, 4]. International studies suggested that 1–25% of pregnant women are exposed to physical violence by intimate partners during pregnancy [5].

Violence against women has serious consequences for their reproductive and sexual health including birth outcomes [6–8]. Violence during pregnancy has been associated with low birth weight, a major cause of infant death in the developing world [3], because stress due to violence raises cortisol levels leading to constriction of the blood vessels, limiting blood flow to the uterus [4].

A systematic review and meta-analysis study revealed that women who reported physical, sexual, or emotional abuse during pregnancy were more likely than nonabused women to give birth to a baby with low birth weight (LBW) [5]. The association between physical violence and LBW remained significant even after adjustment for parity, socioeconomic status, mother's age, and smoking habits [6].

But research linking intimate partner violence during pregnancy to LBW has not been conclusive and was mainly cross-sectional studies. Consequently, how much LBW is attributable to intimate partner violence during pregnancy remained unknown. Most of the research was also conducted in developed countries. In Ethiopia, the average parity of pregnant women is much higher than that in other African countries and no study has been conducted to link intimate partner violence with low birth weight [7]. Thus, we sought to investigate the effects of intimate partner violence during pregnancy period on birth weight of the babies born to women at the four governmental hospitals in Bale Zone, Oromia regional state, Ethiopia.

## 2. Method

### 2.1. Study Setting and Population

A hospital-based case-control study design was conducted in Bale Zone, Southeast Ethiopia, from April 1 to August 30, 2013. This study was conducted at the four government hospitals: Goba, Robe, Delomena, and Ginir. All mothers who gave live births in the study hospitals were eligible for this study. Cases were mothers who gave live births of weight less than 2500 g and controls were mothers who gave live births of weight 2500 g and above. All mothers selected as cases and controls were mothers with singleton and full term births. Additionally, mothers who had serious illness, hypertension, and/or diabetes mellitus were excluded from the study. Seven cases and respective controls' data were excluded because of missing data making the response rate 94%.

For each case there were two controls. Following each case two consecutive controls were included in the study until the required sample sizes were satisfied.

Since the cases (LBW) were rare, all eligible cases fulfilling the inclusion criteria in each hospital were included in the study until the required sample sizes were satisfied within the study period.

### 2.2. Data Collection Procedures

The data were collected by face-to-face interview method using structured and pretested questionnaire. The questionnaire was adopted from the Ethiopian Demographic and Health Survey (EDHS) and Behavioral Surveillance Survey (BSS). It consists of sociodemographic, obstetric, and experiences-of-violence related questions. The same interviewer was used to interview the mother for a case and the respective two consecutive controls. The weight of the newborns was measured within 15 minutes after birth using a balanced Seca scale. Maternal height was measured against a wall height scale to the nearest centimeter. Maternal weight was also measured by beam balance to the nearest kilogram and body mass index (BMI) was subsequently calculated.

The interview and anthropometric measurements were obtained by eight (two in each study hospital) trained midwives and nurses who were working in labor ward.

### 2.3. Data Analysis

The data were analyzed using SPSS for Windows version 20.0 (IBM SPSS Statistics, IBM Corp., New York). Bivariable logistic regression analyses were done to evaluate the association of low birth weight with each construct of intimate partner violence (IPV) (physical violence, psychological violence, and sexual violence) and overall IPV. Multivariable logistic regression analysis was used to control for potential confounding variables. A multivariable analysis was based on multiple logistic regression models. Two multivariable logistic regression models were constructed: one is to see the interaction of the three constructs of IPV, socioeconomic factors, and other maternal factors with the dependent variable low birth weight while the other model is constructed to see the interaction of overall IPV, socioeconomic factors, and other maternal factors with low birth weight. Furthermore, the relationship between the socioeconomic factors (residence, maternal education, maternal occupational status, family monthly income level, and husband's educational and occupational status) and intimate partner violence was evaluated by logistic regression analysis. In order to evaluate the strength of association, both crude and adjusted odds ratios with 95% confidence interval were calculated for exposure to intimate partner violence, socioeconomic factors, and other maternal factors in relation to LBW. Statistical significance was defined as *P* < 0.05.

### 2.4. Operational Definition

#### 2.4.1. Emotional/Psychological Violence

Emotional/psychological violence is defined as being humiliated, insulted, intimidated, or threatened and/or controlling behaviors by a partner.

#### 2.4.2. Physical Violence

Physical violence is defined as being slapped or having something thrown at her that could hurt her, being pushed or shoved, being hit with a fist or something else that could hurt, being kicked, dragged, or beaten up, being choked or burnt on purpose, and/or being threatened with, or actually having, a gun, a knife, or another weapon used on her by an intimate partner.

#### 2.4.3. Sexual Violence

Sexual violence is defined as being physically forced to have sexual intercourse when she did not want to, having sexual intercourse because she was afraid of what her partner might do, and/or being forced to do something sexual that she found humiliating or degrading to her by an intimate partner.

#### 2.4.4. Overall Intimate Partner Violence

Overall intimate partner violence is defined as follows: those mothers who experienced any act of intimate partner violence whether they encounter physical, psychological, or sexual violence during the index pregnancy period.

## 3. Results

In this study, from a total of 408 mothers that we planned to interview, 387 mothers (mothers of 129 cases and 258 controls) completed the interview which made the response rate of 94% for both cases and controls. Fifty-one percent of mothers of LBW babies and 69.4% of mothers of normal birth weight (NBW) babies were in the age range of 21–35 years. The predominant religion was found to be Islam, 66.7% of mothers of cases and 53.9% of mothers of controls. Related to occupational status the majority, 69.8%, of LBW mothers and 45.3% of NBW mothers were housewives. The significant number, 45.7%, of mothers of low birth weight babies was illiterate compared to 15.5% of mothers of normal birth weight babies. Concerning monthly family income among the study population, relatively high percentage of mothers with low birth weight babies, 24.6%, had less than 500 ETB monthly income compared to mothers with normal birth weight babies, 7.8%.

Fifty percent of mothers with LBW babies spaced between present and past pregnancy more than two years compared to 75% of mothers with NBW babies. Almost eighty-eight percent of mothers of cases and 95.3% of mothers of controls weighed more than 50 kg. Measurements of maternal height showed that 84.5% of mothers of cases and 93.8% of mothers of controls were greater than 150 cm tall. Forty-eight percent of mothers of cases and 24.8% of mothers of controls were residing in rural part of the study area. Almost all, 93%, of the study participants were currently married (Table 1).

A total of 387 mothers were interviewed about their experience of any violence during their current pregnancy period. Of them, 100 (25.8%) experienced some violence by their intimate partners during their index pregnancy period. Relatively more mothers of low birth weight infants were abused, 59 (48%), compared with controls, 41 (16.4%). Thirty percent of the mothers of LBW infants had been sexually abused by their partners during their current pregnancy, compared with 7.3% of mothers of the controls. Around forty-one percent of mothers of cases and 10% of mothers of controls had been abused physically by their intimate partners. In addition to physical and sexual violence, 38% of mothers of cases had been psychologically abused by their intimate partners, compared to 11.6% of mothers of controls. Overall, proportionally more mothers in the cases group reported experiences of abuse than in the controls group.

More than half, 54 (14%), of the mothers faced a type of intimate partner violence: the most frequent was being slapped or punched on the face. Seven (1.8%) of the mothers experienced a type of violence less frequent which was not being allowed to enter home or being locked in. Besides the above violence, 46 (11.9%) of the mothers had their contact with friends/family members limited, 44 (11.4%) of the mothers have been criticized by partners for what they were doing, 21 (5.4%) of the mothers were verbally abused by partners somewhere, and 9 (2.3%) of the mothers were threatened with some harmful objects.

Multivariable logistic regression analyses were performed to control confounding variables and to identify the strength of association with significant explanatory variables. In the logistic regression analysis, mothers with no formal education were more likely to encounter physical violence than mothers with advanced educational status (OR = 2.74; 95% CI = 1.38 to 5.47), and housewife mothers were more likely to be physically violated by their partner than employed mothers (OR = 2.859; 95% CI = 1.073 to 7.616). Similarly, mothers living in a family with monthly income less than 500 ETB (OR = 3.35; 95% CI = 1.58 to 7.13) or in the range of 1001–1500 ETB (OR = 2.83; 95% CI = 1.37 to 5.83) were at a higher risk of physical violence compared to mothers living in a family with monthly income greater than 1500 ETB. Psychological violence also significantly increased with residence and family income level. Mothers residing in rural area and with lower income level were associated with increased psychological violence. The relationship between exposure to any type of intimate partner violence and LBW was evaluated after adjusting for residence, maternal education, maternal occupational status, husband's educational and occupational status, family monthly income, maternal body mass index (BMI), antenatal care, and khat chewing and alcohol drinking status of mothers. Therefore, the multivariable logistic regression model indicated that those mothers who suffered acts of any type of intimate partner violence during pregnancy were three times more likely to have a newborn with low birth weight (95% CI: 1.57 to 7.18) (Table 2).

Similarly, the association was examined for subcategories of intimate partner violence (sexual, physical, and psychological) (Table 2). Physical violence during pregnancy was consistently associated with a significant increase in LBW. A slightly higher proportion of mothers of low birth weight infants reported having been abused sexually and psychologically compared with controls, but the difference was not statistically significant.

## 4. Discussion

In this study, 26% of the mothers had experienced intimate partner violence during their index pregnancy period. This result is consistent with other study in Nicaragua where 27% of the mothers had been abused by their intimate partners during their pregnancy [6]. But it is less than the study result in South Carolina where 35% reported intimate partner violence before and during index pregnancy period [7]. The difference might be due to differences in community awareness of violence during pregnancy. This study revealed that relatively more mothers of low birth weight infants were abused compared with controls. This result is supported by other similar studies in which slightly more mothers of low birth weight babies faced intimate partner violence than mothers with normal birth weight babies [2, 5, 6].

Although some studies have found that abuse during pregnancy increases the risk of LBW [1, 4, 7] other studies did not find a significant association [6, 9, 10]. This discrepancy might be due to differences in study populations, sample size, study design, measurements of violence, analytic approaches, and handling of potentially confounding variables.

In our study mothers who suffered from any type of intimate partner violence during pregnancy were more likely to have a newborn with low birth weight. This result is supported by results from hospital-based case-control study in León, Nicaragua, which found that, after adjusting for other known risk factors of low birth weight, partner violence against pregnant women increased the risk of low birth weight by a factor of three [11]. It has also been demonstrated through a systematic review and meta-analyses that the risk of violence during pregnancy increases for both low birth weight and preterm birth [5, 7, 12].

In our study we tried to analyze the association of birth weight for subgroups of intimate partner violence (sexual, physical, and psychological). As a result, physical violence during pregnancy was consistently associated with a significant increase in LBW. This finding is in line with other similar studies where physical violence by intimate partners during pregnancy period was independently associated with low birth weight [4, 6, 13, 14]. While a slightly higher proportion of mothers of low birth weight infants reported having been abused sexually and psychologically compared with controls, the difference was not statistically significant. Other studies demonstrate that statistically significant associations were not seen for those reporting only sexual or only psychological violence during pregnancy [4, 15, 16]. Further, a history of physical intimate partner violence exposure was found to be associated with low infant birth weight in the recent study in South Africa. But neither sexual nor psychological violence exposure yielded significant associations with low birth weight in this study [17].

## 5. Conclusion

The results of the present study indicate that there is an independent effect of overall and physical violence during pregnancy on the birth weight of the offspring. But neither sexual nor psychological violence exposure yielded significant associations with low birth weight in our study.

Health service providers should be aware of the impact of intimate violence on pregnant women and on their newborns and try to identify women at risk using the knowledge of maternal characteristics statistically associated with violence in pregnancy.

Longitudinal studies would also be valuable in addressing the causal mechanisms between intimate violence and low birth weight.

## Supplementary Material

Supplementary material contains the structured questionnaire in Amharic and English languages, available online for readers.

## Figures and Tables

**Table 1:** Distribution of mothers by sociodemographic characteristics in Bale Zone, Oromia regional state, August 2013.

Variables	LBW		NBW		Total	
	Number	%	Number	%	Number	%
Age group (years)						
<20	52	40.3	56	21.7	108	28.0
21–35	66	51.2	179	69.4	245	63.3
>35	11	8.5	23	8.9	34	8.7
Weight group (kg)						
<50	16	12.4	12	4.7	28	7.2
>50	113	87.6	246	95.3	359	92.8
Height (Cm)						
≤150	20	15.5	16	6.2	36	9.3
>150	109	84.5	242	93.8	351	90.7
Religion						
Muslim	86	66.7	139	53.9	225	58.1
Orthodox	38	29.5	100	38.8	138	35.7
Protestant	5	3.9	14	5.4	19	4.9
Catholic	0	0	5	1.9	5	1.3
Ethnicity						
Oromo	103	79.8	183	70.9	186	73.9
Amhara	14	10.9	64	24.8	78	20.2
Others	12	9.3	11	1.3	23	5.9
Marital status						
Married	116	89.9	243	94.2	359	92.7
Others	13	10.1	15	5.8	28	7.3
Residence						
Urban	67	51.9	194	75.2	261	67.4
Rural	62	48.1	64	24.8	126	32.6
Head of household						
Male	117	90.7	233	90.3	350	90.4
Female	12	9.3	25	9.7	37	9.6
Maternal occupation						
Employed	10	7.8	40	15.5	50	12.9
Housewife	90	69.8	117	45.3	207	53.4
Farmer	8	6.2	31	12.0	39	10.0
Merchant	12	9.3	63	24.4	75	19.3
Daily laborer	9	7.0	7	2.7	16	4.3
Monthly income (ETB)						
≤500	31	24.6	20	7.8	51	13.3
501–1000	40	31.7	49	19.1	89	23.2
1001–1500	15	11.9	39	15.2	54	14.1
>1500	40	31.7	149	58	189	49.3
Maternal education						
Illiterate	59	45.7	40	15.5	99	25.6
Read and write only	2	1.6	5	1.9	7	1.8
Primary (1–8)	49	38.0	109	42.2	158	40.8
Secondary (9–12)	14	10.9	81	31.4	95	24.5
Tertiary	5	3.9	23	8.9	28	7.2

**Table 2:** Sexual, physical, and psychological and all intimate violence during pregnancy and the risk of delivering a low birth weight infant among mothers in Bale Zone, Oromia regional state, August 2013.

Violence	LBW	NBW	COR [95% CI]	*P* value	AOR^*∗*^ [95% CI]	*P* value
Sexual violence						
Yes	38	16	5.4 [2.92, 10.08]	<0.001	1.5 [0.43, 5.54]	0.513
No	84	228	1		1	

Physical violence						
Yes	50	25	6.2 [3.56, 10.66]	<0.001	8.5 [3.36, 21.73]	<0.001
No	73	225	1		1	

Psychological violence						
Yes	47	29	4.7 [2.77, 8.01]	<0.001	1.03 [0.29, 3.75]	0.957
No	76	221				

Over all types of violence						
Yes	59	41	4.7 [2.89, 7.65]	<0.001	3.4 [1.57, 7.18]	0.002
No	64	209	1		1	

^**∗**^Adjusted for birth interval, maternal height, BMI, maternal weight, antenatal care follow-up, and khat chewing status of the mother.
