# Supplementary material for: A Case-Control Study on Intimate Partner Violence during Pregnancy and Low Birth Weight, Southeast Ethiopia
Source: Obstet Gynecol Int. 2015 Dec 20;2015:394875. doi: 10.1155/2015/394875 (PMC4698783; doi:10.1155/2015/394875)
Supplement: Supplementary file 1 — Supplementary material contains the structured questionnaire in Amharic and English languages, available online for readers. [file 394875.f1.pdf]

1. Annexes

Annex I- Structured Questionnaire in English

Inclusion criteria

1. Being term baby
2. Mothers have no history of diabetes mellitus

Anthropometric measurement

Variable	Measurement	Remark
Gestational age	**/_________/ Weeks**	
Neonatal weight	**/_________/Grams**	
Maternal weight	**/_________/Kg**	
Maternal height	**/_________/Meters**	

Part I: Socio-EconomicVariables

No	Questions	Response Options	Skip
101	Head of house hold	1. Male<br>2. Female	
102	Marital status	1. Married<br>2. Divorced<br>3. Separated<br>4. Single<br>5. Widowed	
103	Religion	1. Orthodox<br>2. Protestant<br>3. Muslim<br>4. Catholic<br>9. Others (specify) /____________ /	

104	Ethnicity	1. Amhara<br>2. Tigrie<br>3. Oromo<br>4. Guragie<br>9. Others (specify) ___________	
105	Residential area	1. Urban<br>2. Rural	
106	Total family size of the house hold	Write in number /_________/	
107	Total number of < 5 children	Write in number / _________/	
108	Have you attended formal education?	1. Yes<br>2. No	
109	If **yes** to **Q108,** what is your highest grade completed?	Grade ____________	
110	If **No** to **Q108,** are you able to read and write?	1. Yes<br>2. No	
111	Educational status of the husband	1. Illiterate<br>2. Read &write only<br>3. Highestgrade completed___________	
112	Maternal occupational status<br>(***more than one response is possible***)	1. Housewife<br>2. Farmer<br>3. Private employee<br>4. Merchant<br>5. Daily laborer<br>6. Gov't employee<br>9. Other (specify) /________________/	
113	Occupational status of the husband (more than one response is possible)	1. Farmer<br>2. Merchant<br>3. Private employee	

		4. Daily laborer<br>5. Gov't employee<br>9. Other (specify) /______________/	
114	Monthly income of the family	____________________birr/month<br><br>____________________birr/year	
115	Who decides how the money you earn will be used?	1. Respondent alone<br>2. Respondent and husband/partner jointly<br>3. Husband/partner alone<br>4. Someone else(specify____________)	

Part II: Maternal Characteristics

116	Mother's age in years	Year completed /__________/	
117	Age at first birth	/_____/ Years	
118	Age when the youngest child was born	/_____/ Years	
119	Total number of pregnancy	/ _______/ in number	
120	Total number of live births?	/ _______/ in number	
121	Have you visited health facility for ANC for current pregnancy?	1. Yes<br>2. No	►
122	At what months of the current pregnancy you started ANC?	1. At ________ months<br>2. Don't know/not sure	
123	How many times you visited health facility for ANC?	/________/ times	
124	Where did you attend the ANC during your pregnancy?	1. Health post<br>2. Health center<br>3. Gov't hospital<br>4. Private hospital<br>5. Private clinic<br>6. NGOs clinic<br>9. Others(specify) /__________/	

125	During your ANC visit(s), are you informed about signs of pregnancy complications?	1. Yes<br>2. No	
126	If*yes* for***Q125***, which complications? (***more than one response is possible***)	1. Vaginal bleeding<br>2. Vaginal gush of fluid.<br>3. Severe headache<br>4. Blurred vision<br>5. Fever<br>6. Abdominal pain<br>9. Other /________________/	
	Have encountered any of the mentioned complications?	1. Yes<br>2. No	
	If **yes for Q-----,** which complications? (***more than one response is possible***)	1. Vaginal bleeding<br>2. Vaginal gush of fluid.<br>3. Severe headache<br>4. Blurred vision<br>5. Fever<br>6. Abdominal pain<br>9. Other /________________/	
127	Have you taken any deworming drugs during your pregnancy?	1. Yes<br>2. No<br>3. Do not know/not sure	
128	Is there any history of abortion?	1. Yes<br>2. No	
129	If **yes for Q128,** how many times?	/_________/times	
130	If **yes for Q128,** which type of abortion was it?	1. Spontaneous abortion<br>2. Induced abortion	
130	If ***yes*** for ***Q128,*** by whom the abortion was conducted?	1. Myself/relatives/friend<br>2. TBA<br>3. TTBA<br>4. Health workers	
131	Have you ever chewed Khat?	1. Yes<br>2. No	
132	If **yes for Q131,** have you chewed khat in the	1. Yes<br>2. No	

	last 12 months?		
133	If **yes for Q132,** how frequent have chewed khat?	_________/ day<br>_________/ week<br>__________/month	
134	Have you ever smoked cigarette	1. Yes<br>2. No	
135	If **yes for Q134,** have you smoked in the last 12 months?	1. Yes<br>2. No	
136	If **yes for Q135,** How many cigarettes have smoked per day?	___________/day	
137	Have ever drunk alcohol?	1. Yes<br>2. No	
138	If **yes for Q137,** have you drunk alcohol in the last 12 months?	1. Yes<br>2. No	
139	Which type?<br>(***More than one answer is possible.)***	1. Tela<br>2. Tej<br>3. Areqe<br>4. Beer/draft<br>5. Wine<br>6. Whisky<br>7. Others (specify)_______	

Part III: Violence /Abuse

131	Have you had a relationship over the past 12 months?	1. Yes<br>2. No
132	How many years have you been in this relationship	/_______/ months(***only for <1 year***)<br>/_______/ years
133	Are you currently in a relationship?	1. Yes<br>2. No
134	Were /are you happy in your relationship?	1. Yes<br>2. No
135	If the relationship ends, How many months has it been since ended?	/__________/ months
136	Was your relationship ended with your	1. Yes<br>2. No

	agreement?	
137	Over the last 12 months have you been verbally abused or insulted on the street, on public transport or in other public areas by your partner?	1. Yes<br>2. No<br>3. Do not remember
138	If **yes** to **Q# 137**, how many times?	/_________________/times
139	Over the past 12 months has your partner threatened you with a weapon, a dangerousobject (knife, bat ...) or has tried to kill you or strangle you?	1. Yes<br>2. No<br>3. Donot remember
140	If **yes** to **Q# 139,** how many times?	/_________________/times
141	Over the last 12 months, has your partner forced you to undergo sexual touching, or having sexual contact against yourwill?	1. Yes<br>2. No<br>3. Do not remember
142	If **yes** to **Q# 131**, how many times?	/_________________/times

Thinking about your partner or last partner, would you say that over the last 12 months, he

143	Has tried to limit the contact you have with your friend(s) or family members?	1. Yes<br>2. No<br>3. Does not know
144	If **yes** to **Q# 143,** how many times?	/_________________/times
145	Has sworn at you, criticized, ridiculed you for what you were doing or saying?	1. Yes<br>2. No
146	If **yes** to **Q# 145**, how many times?	/_________________/times
147	Has stopped talking to you, totally refused to discuss things with you?	1. Yes<br>2. No<br>3. Do not remember
148	If **yes** to **Q# 147**, how many times?	/_________________/times
149	Thrown something at you, shaken you or grabbed you suddenly?	1. Yes<br>2. No<br>3. Do not remember
150	If **yes** to **Q# 149**, how many times?	/_________________/times

151	Scratched you, pinched you, bitten you or pulled your hair?	1. Yes<br>2. No<br>3. Do not remember
152	If **yes** to **Q# 151**, how many times?	/_________________/times
153	Slapped you, punched or kicked you, hit you with something that hurt you?	1. Yes<br>2. No<br>3. Do not remember
154	If **yes** to **Q# 153**, how many times?	/_________________/times
155	Prevented from entering your home, locked you up, locked you out, or when in thecar, left you by the roadside?	1. Yes<br>2. No<br>3. Do not remember
156	If **yes** to **Q# 155,** how many times?	/_________________/times

Part IV: Environmental Health Conditions

401	What is your main source of drinking water?	1. Piped in to dwelling<br>2. Public tap<br>3. Rain water<br>4. Protected spring<br>5. Protected well<br>6. Unprotected spring<br>7. Unprotected well<br>8. River/stream<br>9. Tube well/borehole with hand pump<br>10. Other (specify) / ____________/	
402	Average household water use per day per person for drinking, cooking and personal hygiene	__________in liters	
403	Average time taken to and from the nearest water point (including waiting time)	1. /__________/ In minutes<br>2. Don’t know	
404	What is the method of water storage in the household?	1. Covered containers<br>2. Open containers<br>3. Constricted neck/end<br>9. Others (specify) /______________/	

405	How could you draw water from the storage?	1. Pouring<br>2. Deeping<br>9. Others (specify) /_______________/	
406	Do you treat water in any way to make it safer?	1. Yes<br>1. No	
407	If **yes** to **Q# 406**, what activities do you practiced?	1. Boiling<br>2. Chemical treatments<br>3. Filtering using cloth<br>4. filtering using sand, gravel or ceramics<br>5. Sun radiation treatment<br>6. Sedimentation<br>9. Others (specify) /_______________/	
408	Have you got education by health workers on how you can make water safe for drinking?	1. Yes<br>2. No<br>1. Not known/not sure	
409	Have you got advice from health workers about the advantages of washing hands by means of soap or ash?	1. Yes<br>2. No<br>9. Others (specify) /_______________/	
410	Do you have latrine?	1. Yes<br>2. No	
411	If **yes** to **Q# 1410**, type of latrine the households use?	1. Private traditional pit latrine<br>2. Private wooden slab latrine<br>3. Private cement slab latrine<br>4. Private VIP latrine<br>5. Shared wooden slab latrine<br>6. Shared VIP latrine<br>7. Flash to sewerage system<br>9 Other (Specify) _____________	
412	If **No** to **Q# 410**, where does the family defecate?	1. At the back yard<br>2. Open field<br>9. Others (specify) /_______________/	
413	What happens to the stools of young children (0-3 years) when they do not use the latrine or toilet facilities?	1. Throw in to toilet/latrine<br>2. Throw outside the yard<br>3. Buried in the yard<br>4. Not disposed or left on the ground<br>9 Other(specify) /________________/	

414	Mainly, how do the household dispose solid wastes?	1. In a private pit<br>2. Common pit<br>3. Composting<br>4. Burring<br>5. Burning<br>6. Open field disposal<br>7. By other organized agents<br>9 Other (specify) /_____________/	
415	Mainly, when do you wash your hands? (***More than one answer is possible***)	1. After visiting the latrine<br>2. Before preparing the food<br>3. Before serving food<br>4. After eating<br>5. After cleaning child faeces<br>9. Other(Specify) _____________	
416	How do you wash your hand?	1. Using water only<br>2. Using soap and water<br>3. Using ash with water<br>9. Others(specify) __________	
417	How many rooms for use by the member of your household? (***Exclude kitchen and store***)	________in number	
418	Presence of windows	1. Yes opened<br>2. Yes but not opened<br>3. No window/s<br>9. Other (specify)__________________	
419	Do you have separate room which is used as Kitchen?	1. Yes<br>2. No	
420	If you have livestock, do you have separate room for them?	1. Yes<br>2. No	
421	What type of fuel do you mainly use for cooking?	1. Wood 2. Kerosene<br>3. Electricity 4. Animal dung<br>9. Other(specify) _____________	

Annex II. Structured Questionnaire in Amharic

አነስተኛ ክብደት ያላቸዉ ህፃናትን በሚወልዱ እናቶች ላይ ለሚደረግ ጥናት የመረጃ መሰብሰቢያ ቅፅ

በጥናቱ የሚካተቱ

- የእርግዝና ጊዜያቸዉ ከ 37 ሳምንት በላይ የሆናቸዉ ህፃናት (Term Babies)
- የስኳር ህመም የሌለባቸዉ እናቶች (Non Diabetic Mothers)

አካላዊ ልኬት(Anthropometric Measurement)

የሚለካዉ አይነት	ልኬት	ምርመራ
የእርግዝናጊዜ	/__________/ ወራት	
የህጻኑ/ንዋክብደት	/__________/ግራም	
የእናትዮዋ ክብደት	/__________/ኪ..ግ	
የእናትዮዋ ቁመት	/__________/ሜትር	

ክፍል አንድ: ማህበራዊና ኢኮኖሚያዊ ሁኔታ

ተ ቁ	ጥያቄወች	የተሰጡ መልሶች
101	የምትኖሪበትን ቤት የሚያስተዳድረዉ	3. ወንድ 2.ሴት
102	የጋብቻ ሁኔታ	6. ያገባች<br>7. የተፋታች<br>8. ተለያይተዉ የሚኖሩ<br>9. ያላገባች<br>10. የሞተባት
103	ሃይማኖትሽ ምንድነው?	1. ሙ ስሊም<br>2. ኦርቶዶክስ<br>3. ፕሮቲስታንት<br>4. ካቶሊክ<br>9. ሌላ(ይገለፅ) /______________/
104	ብሄርሽ ምንድን ነዉ?	5. ኦሮሞ<br>6. አማራ<br>7. ትግሬ<br>8. ጉራጌ<br>9. ሌላ (ይገለፅ) /______________/
105	የመኖሪያ ቦታ	3. ከተማ 2.ገጠር
106	የቤተሰብ አባላት ቁጥር	በቁጥር ይጻፍ /__________/
107	ከአምስት ዓመት በታች (<5) የሆኑ ልጆች ቁጥር	በቁጥር ይጻፍ /__________/
108	መደበኛ ትምህርት ተከታትለሻል?	1. አዎ 2. አልተከታተልኩም
109	ለ**ጥ.ቁ 108** መልስ አዎ ከሆነ፣ያጠናቀቅሽዉ ክፍል	________ኛ ክፍል
110	ለ**ጥ.ቁ 108** መልስ አልተከታተልኩም ከሆነ፣ማንበብና መፃፍ ትችያለሽ?	1. አዎ 2.አልችልም
111	የባለቤትሽ የትምህርት ደረጃስንት ነዉ?	1. ያልተማረ<br>2. መፃፍ እና ማንበብ ብቻ የሚችል<br>3. ያጠናቀቀዉ ክፍል/________/
		1. የቤት እመቤት

112	ስራሽ ምንድን ነዉ? / ከአንድ በላይ መልስ ይቻላል/	2. ግብርና<br>3. የግል ተቀጣሪ<br>4. ንግድ<br>5. የቀን ስራ<br>6. የመንግስት ተቀጣሪ<br>9. ሌላ(ይገለፅ) /______________/
113	የባለቤትሽ ሰራ ምንድን ነዉ? / ከአንድ በላይ መልስ ይቻላል/	1. ግብርና<br>2. ንግድ<br>3. የመንግስት ተቀጣሪ<br>4. የግል ተቀጣሪ<br>5. የቀን ስራ<br>9. ሌላ(ይገለፅ) /____________/
114	ወርሃዊ ገቢያችሁ ስንት ይሆናል?	1. ________ ብር/ ወር<br>2. ________ ብር/ አመት
115	ወርሃዊ ገቢያችሁን እንዴት መጠቀም እንዳለባችሁ የሚወስነዉ ማነዉ?	5. እኔ ብቻ<br>6. ባለቤቴና እኔ በጋራ<br>7. ባለቤቴ ብቻ<br>9. ሌላ/ይገለፅ /________________/

ክፍል ሁለት : የእናትዮዋን ሁኔታ በተመለከተ

201	እድሜ በአመት	ያጠናቀቀችዉ አመት ይፃፍ /_____/
202	ይህ የተወለደዉ/ችዉ ህፃን የመጀመሪያሽ ነዉ/ች?	1. አዎ → **ቁ. 207 ይለፉ**<br>2. አይደለም
203	የመጀመሪያ ልጅሽን የወለድሽዉ በስንት አመትሽ ነበር?	/______/ አመቴ
204	ከዚህ በፊት የወለድሽዉን ልጅ ስትወልጅ ስንት አመትሽ ነበር?	/______/ አመቴ
205	እስከ አሁን ድረስ ስንት ጊዜ አርግዘሻል?	/______/ በቁጥር ይፃፍ
206	አጠቃላይ በህይወት የተወለዱ ልጆች ብዛት ስንት ነው?	/ ________/ በቁጥር ይፃፍ
207	ይህን/ችን ህጻን ከማረገዝሽ በፊት(በ12 ወራት ዉስጥ) ክብደትሽ ስንት ነበር?	1. /_________/ ኪ.ግ<br>2. አላስታዉሰዉም
208	ለዚህኛዉ እርግዝና የቅድመ ወሊድ ክትትል ታደርጊ ነበር?	1. አዎ<br>2. አልተከታተልኩም **ጥ.ቁ → 214 ይለፉ**
209	በስንተኛዉ ወር ላይ መከታተል ጀመርሽ?	1. በ______ኛዉ ወር ላይ<br>2. አላስታዉሰዉም
210	ለቅድመ ወሊድ ክትትል ለምን ያህል ጊዜ ወደ ጤና ተቋም ሄድሽ?	/_________/ ጊዜ
211	የቅድመ ወሊድ ክትትል ያደረግሽዉ የት ነበር?	7. ጤና ኬላ<br>8. ጤና ጣቢያ<br>9. ከመንግስት ሆስፒታል<br>10. ከግል ሆስፒታል<br>11. ከግል ክሊኒክ<br>12. መንግስታዊ ካልሆነ ድርጅት ክሊኒክ<br>10. ሌላ ይገለፅ /____________/
212	የቅድመ ወሊድ ክትትል በምታደርጊበት ወቅት ከእርግዝና ጋር የተያያዙ የጤና ችግር ምልክቶች ተነግሮሽ ነበር?	3. አዎ<br>4. የለም
213	ለጥያቄ ቁ 212 መልስሽአዎ ከሆነ፣ከሚከተሉት የትኛዉ ተነገረሽ? (ከአንድ በላይ መልስ ይቻላል)	7. የብልት መድማት<br>8. የብልት ፈሳሽ<br>9. ከባድ የራስ ምታት<br>10. የእይታ ብዥታ<br>11. ትኩሳት<br>12. የሆድ ህመም<br>9. ሌላ ይገለፅ /____________/
214	ከእርግዝና ጋር የተያያዙ የጤና ችግሮች አጋጥሞሽ ነበር?	1. አዎ<br>2. የለም

215	*ለጥያቄ ቁ214 መልሰሽ አዎ ከሆነ ከላይ ከተዘረዘሩት በአንዱ ላይ የተከሰቱት የትኞቹ ናቸዉ?*(ከአንድ በላይ መልስ ይቻላል)	1. የብልት መድማት<br>2. የብልት ፈሳሽ<br>3. ከባድ የራስ ምታት<br>4. የእይታ ብዥታ<br>5. ትኩሳት<br>6. የሆድ ህመም<br>9. ሌላ ይገለፅ /____________/
216	በእርግዝና ወቅት ጸረ-ትላትል መድሃኒት ወስደሽ ታዉቂያለሽ?	4. አዎ<br>5. አልወሰድኩም<br>6. አላስታዉስም
217	*ጽንስ ሉቋርጠሽ ወይም ዉርጃ አካሂደሽ ታዉቂያለሽ?*	1. አዎ<br>*2.* የለም → [illegible]*ያቄ ቁ220 ይለፉ*
218	*ለጥያቄ ቁ217 መልሰሽ አዎ ከሆነ ለምን ያህል ጊዜ*	/__________/ ጊዜ
219	*የዉርጃዉ ዓይነት ምን ነበር?*	**1.** በራሱ ጊዜ<br>**2.** በማነሳሳት/ሆንተብሎ
220	*ጽንሱን ያቋረጥሽዉ/ያስወረድሽ በማን ነበር?*	**1.** በራሴ/በዘመድ/በጓደኛ<br>**2.** በልምድ አዋላጅ<br>**3.** በሰለጠነ የልምድ አዋላጅ<br>**4.** በጤና ባለሙያ
221	*ጫት ቅመሽ ታዉቂያለሽ?*	1. አዎ 2. የለም → *ጥ.ቁ224 ይለፉ*
222	*ለጥያቄ ቁ221 መልሰሽ አዎ ከሆነ, ባለፉት 12 ወራት ዉስጥ ቅመሽ ታዉቂያለሽ?*	1. አዎ<br>2. የለም
223	*ለጥያቄ ቁ221 መልሰሽ አዎ ከሆነ, ለምን ያህል ጊዜ ትቅሚያለሽ?*	/__________/ በቀን<br>/__________/ በሳምንት<br>/__________/ በወር
224	ሲጋራ አጭሰሽ ታዉቂያለሽ?	1. አዎ<br>2. የለም → [illegible]*ያቄ ቁ227 ይለፉ*
225	*ለጥያቄ ቁ224 መልሰሽ አዎ ከሆነ, ባለፉት 12 ወራት ዉስጥ አጭሰሽ ታዉቂያለሽ?*	1. አዎ<br>2. የለም
226	*ለጥያቄ ቁ224 መልሰሽ አዎ ከሆነ, በቀን ስንት ሲጋራ ታጨሻለሽ?*	በቀን/__________/ ሲጋራ
227	የአልኮል መጠጥ ጠጥተሽ ታዉቂያለሽ?	1. አዎ<br>2. የለም → [illegible]*ደ ክፍል ሦስት ይለፉ*
228	*ለጥያቄ ቁ227 መልሰሽ አዎ ከሆነ, ባለፉት 12 ወራት ዉስጥ ጠጥተሽ ታዉቂያለሽ?*	1. አዎ<br>2. የለም
229	*ለጥያቄ ቁ 227 መልሰሽ አዎ ከሆነ, የጠጣሽዉ የአልኮል ዓይነት?(ከአንድ በላይ ምላሽ ይቻላል)*	1. ጠላ<br>2. ጠጅ<br>3. አረቂ<br>4. ቢራ/ድራፍት<br>5. ወይን<br>6. ዉስኪ<br>9. ሌላ (ይጠቀስ) _________

ክፍል ሦስት : ፆታዊ ጥቃትን በተመለከተ

301	ባለፉት 12 ወራት ዉስጥ ፆታዊ ግንኙነት ነበረሽ?	3. አዎ 2. አልነበረኝም
302	በግንኙነቱ ለምን ያህል ጊዜ ቆያችሁ?	/________/ ወራት
303	ባሁኑ ወቅትፆታዊ ግንኙነት አለሽ?	3. አዎ 2. የለኝም
304	በዚህ ግንኙነት ደስተኛ ነሽ?	1. አዎ 2.አይደለሁም
305	ግንኙነት አቁማችሁ ከሆነ፣ካቆማችሁ ምን ያህል ጊዜ ሆናችሁ?	/____________/ወራት
306	ግንኙነት አቁማችሁ ከሆነ ግንኙነቱ የተቋረጠዉ ባንች ስምምነት ነዉ?	1. አዎ

		2. አይደለም
307	ባለፉት 12 ወራት ዉስጥ መንገድ ላይ ወይም በትራነስፖርት ቦታወች ላይ በባለቤትሽ/ጓደኛሽ ተሰድበሽ ታዉቂያለሽ?	4. አዎ<br>5. ተሰድቤ አላዉቅም<br>6. አላስታዉስም
308	መልስሽ አዎ ከሆነለምን ያህል ጊዜ?	/________/ ጊዜ
309	በጩቤ፤በዱላ ወይም በሌላ የጦር መሳሪያ ሊገድልሽ ሞክሮወይም አስፈራርቶሽ ነበር?	4. አዎ<br>5. የለም<br>6. አላስታዉስም
310	መልስሽ አዎ ከሆነለምን ያህል ጊዜ?	/________ / ጊዜ
311	ከፍላጎትሽ ዉጭ የግብረ-ስጋ ግንኙነት እንድትፈፅሚ አስገድዶሽ ያዉቃል?	1. አዎ<br>2. አያዉቅም<br>3. አላስታዉስም
312	መልስሽ አዎ ከሆነለምን ያህል ጊዜ?	/________/ ጊዜ
313	ከቤተሰብ ወይም ጎደኞችሽ ጋር ያለሽን ግንኙነት እንድትገድቢ አስገድዶሽ ያዉቃል?	1. አዎ<br>2. አያዉቅም<br>3. አላስታዉስም
314	መልስሽ አዎ ከሆነ ለምን ያህል ጊዜ?	/________/ ጊዜ
315	የምትሰሪዉን ወይም የምትናገሪዉን የማጣጣል ወይም የመተቸት ባህሪ ነበረዉ?	3. አዎ<br>4. አልነበረዉም<br>5. አላስታዉስም
316	መልስሽ አዎ ከሆነለምን ያህል ጊዜ?	/________/ ጊዜ
317	ካንቺ ጋር ንግግር ወይም ዉይይት አቁሞ ነበር?	1. አዎ<br>2. አልነበረዉም<br>3. አላስታዉስም
318	መልስሽ አዎ ከሆነለምን ያህል ጊዜ?	/________/ ጊዜ
319	ወርዉሮብሽ ወይም እጅሽን ጠምዝዞሽ ያዉቃል?	3. አዎ<br>4. አያዉቅም<br>5. አላስታዉስም
320	መልስሽ አዎ ከሆነለምን ያህል ጊዜ?	/________/ ጊዜ
321	ቆንጥጦሽ፡ቧጭሮሽ ወይም ፀጉርሽንስቦሽ ያዉቃል?	1. አዎ<br>2. አያዉቅም<br>3. አላስታዉስም
322	መልስሽ አዎ ከሆነለምን ያህል ጊዜ?	/________/ ጊዜ
323	በጥፊ፤በቦክስ፤በእርግጫ ወይም በሌላ ሊጎዳ በሚችል ነገር መቶሽ ያዉቃል?	1. አዎ 2. የለም<br>3. አላስታውስም
324	መልስሽ አዎ ከሆነ ለምን ያህል ጊዜ?	/________/ ጊዜ
325	ወደ ቤትሽ እንዳትገቢ፤ ከቤት ዉስጥ ወይም ዉጭ እያለሽ ዘግቶብሽ ወይም ከመኪና አስወርዶ መንገድ ላይ ትቶሽ ሂዶ ያዉቃል?	1. አዎ 2. የለም<br>3. አላስታዉስም

326 መልስሽ አዎ ከሆነ ለምን ያህል ጊዜ? /________/ ጊዜ

ክፍል አራት፡ የአካባቢያዊ የጤና ሁኔታ

401	ለመጠጥነት የምትጠቀሙት የዉኃ ምንጫችሁ ምንድን ነዉ ?	1. በመኖሪያ ግቢያችን ካለ ቧንቧ<br>2. ከህዝብ የቧንቧ ዉኃ/ቦኖ<br>3. የዝናብ ዉኃ<br>4. ከተጠበቀየምንጭዉኃየጥልቅ ጉድጉድ<br>5. ከታጠረ/ከተጠበቀየኩሬ ዉኃ<br>6. ካልታጠረ/ካልተጠበቀየጥልቅ ጉድጉድ ዉኃ<br>7. ካልታጠረ/ካልተጠበቀየኩሬ ዉኃ<br>8. ከወራጅ ወንዝ<br>10. ከዘመናዊ የጥልቅ ጉድጉድ ዉኃ<br><br>9. ሌላ (ይጠቀስ)/ ____________/
402	አማካይ የቤተሰብ የዉኃ ፍጆታ በቀን ምን ያህል ይሆናል?	___________ (በሊትር)
403	ከቤታችሁ ዉኃ እስከምትቀዱበት ድረስ ምን ያህል ጊዜ ይወስዳል? (የመጠበቂያ ጊዜዉን ጨምሮ)	1. ___________ (በደቂቃ)<br>2. አላዉቀዉም
404	ለዉኃ ማጠራቀሚያነት የምትጠቀሙት ምንድን ነዉ?	1. የሚከደኑ ማጠራቀሚያዎችን<br>2. ክፍት ማጠራቀሚያዎችን<br>3. አንገተ ጠባብ ማጠራቀሚያዎችን<br>9. ሌላ (ይጠቀስ) /_____________/
405	ዉኃ ከማጠራቀሚያዉ የምትቀዱት እንዴት ነዉ?	3. በማፍሰስ<br>4. በመጥለቅ<br>9. ሌላ (ይጠቀስ)/______________/
406	የመጠጥ ዉኃን ጤናማ ለማድረግ ዉኃን የምታክሙበት ዘዴ አላችሁ?	2. አዎ<br>3. የለም
407	ለጥያቄ ቁ 406 መልስሽ አዎ ከሆነ የትኞቹን ዘዴዎች ትጠቀማላችሁ?	7. ማፍላት<br>8. በኬሚካል ማከም<br>9. በጨርቅ በማጣራት<br>10. በአሸዋ፤ጠጠር/ሸክላበማጣራት<br>11. በጽኃይ ብርሃን በማከም<br>12. በማጥለል<br>9. ሌላ (ይጠቀስ)/______________/
408	የመጠጥ ዉኃ እንዴት ጤናማ እንደሚደረግ በጤና ባለሙያ ትምህርት ተሰጥቶአችሁ ያዉቃል?	3. አዎ<br>4. የለም
409	እጅን በሳሙና ወይም በአመድ የመታጠብን ጥቅም ከጤና ባለሙያ ምክር አግኝታችሁ ታዉቃላችሁ?	3. አዎ<br>4. የለም
410	ሽንት ቤት አላችሁ?	1. አዎ 2. የለም
411	ለጥያቄ ቁ 410 መልስሽ አዎ ከሆነ፤ምን ዓይነት ነዉ?	8. የግል ክፍት ጉድገድ<br>9. የግል ባለእንጨት መረባረብ<br>10. የግል ከሲሚንቶ የተሠራ<br>11. የግል የተሻሻለ ባለማናፈሻ<br>12. የጋራ ባለእንጨት መረባረብ<br>13. የጋራ የተሻሻለ ባለማናፈሻ<br>9. ሌላ (ይጠቀስ) _____________
412	ለጥያቄ ቁ 410 መልስሽ የለም ከሆነ፤የቤተሰቡአባላት የሚጸዳዱት የት ነዉ?	3. በተከለለ ቦታ<br>4. ሜዳ ላይ<br>9. ሌላ (ይጠቀስ) _____________
413	ሽንት ቤት መጠቀም የማይችሉ ህጻናትከተጸዳዱ በኃላ ሰገራቸዉን ምን ታደርጉታላችሁ ?	5. ወደ ሽንት ቤት ይጣላል/ይወረወራል<br>6. ከግቢ ዉጭ ይጣላል/ይወረወራል<br>7. ግቢ ዉስጥ ይቀበራል<br>8. በተጸዳዱበት ቦታ ይተዋል/ይቀራል

		9. ሌላ (ይጠቀስ) _____________
414	የቤተሰቡ አባላት ደረቅ ቆሻሻን የሚያስወግደዉ የት ነዉ?	1. የግል ደረቅ ቆሻሻ ማጠራቀሚያ<br>2. የጋራ ደረቅ ቆሻሻ ማጠራቀሚያ<br>3. ለፍግነት/ማዳበሪያነትይዉላል<br>4. መቅበር/ማቃጠል<br>5. ሜዳ ላይ<br>6. በተደራጁ ማህበራት<br>9. ሌላ (ይጠቀስ) _____________
415	በአብዛኛዉ እጅሽን የምትታጠቢዉ መቼ ነዉ? (ከአንድ በላይ ምላሽ ይቻላል)	1. ከሽንት ቤት መልስ<br>2. ከምግብ ዝግጅት በፊት<br>3. ከምግብ ከማቅረብ በፊት<br>4. ከመመገብ በፊት<br>5. ከመመገብ በኃላ<br>6. ህጻናትን ካጸዳዳሁ በኃላ<br>9. ሌላ (ይጠቀስ) ______________
416	እጅሽን የምትታጠቢዉ በምንድን ነዉ?	1. በዉኃ ብቻ<br>2. በዉኃ እና በሳሙና<br>3. በዉኃ እና በአመድ<br>9. ሌላ (ይጠቀስ) ___________
417	የምትኖሩበት ቤት ስንት ክፍሎች አሉት? (እቃቤትና ኩሽና ሳይጨምር)	_________ (በቁጥር)
418	የምትኖሩበት ቤት መስኮት አለዉ?	1. አዎ 2.የለዉም
419	ለጥያቄ ቁ 418 መልስሽ አዎ ከሆነ የመስኮቶች ሁኔታ?	1. እንከፍተዋለን 2.አንከፍተዉም
420	ለኩሽናነት የሚያገለግል የተለየ ክፍል አላችሁ?	1. አዎ 2.የለም
421	ለእንስሳት መኖሪያነት የሚያገለግል የተለየ ክፍል አላችሁ?	1. አዎ 2. የለም
422	በአብዛኛዉ ለምግብ ማብሰያነት የምትጠቀሙት ምንድን ነዉ?	1. እንጨት<br>2. ጋዝ<br>3. ኤሌክትሪክ<br>4. ኩበት<br>9. ሌላ (ይጠቀስ) ______________

የጠያቂው ስም____

ቃለ መጠይቁ የተደረገበት ቀን ______ /______/ 2005 ፊርማ _________________

የተቆጣጣሪው ስም _____________________________/ ፊርማ___________________________

- *የመጠይቁ መለያ ቁጥር* _____________________
- *የሆስፒታሉ ስም* ________________________
- *መጠይቁ የተደረገበት ሰዓት*_____________________
